# Ethics domains in full health technology assessment reports: an attempt to begin mapping the field

**DOI:** 10.1017/S026646232400480X

**Published:** 2025-04-08

**Authors:** Hannes Kahrass, Antje Schnarr, Clovis Mariano Faggion, Marcel Mertz

**Affiliations:** 1 Institute for Ethics, History and Philosophy of Medicine, Hannover Medical School, Hannover, Germany; 2 Department of Periodontology and Operative Dentistry, University Hospital Münster, Münster, Germany

**Keywords:** Health Technology Assessment, ELSO, Ethics, Meta-research

## Abstract

**Introduction:**

Health technology assessment (HTA) reports are written for healthcare decision makers, particularly in relation to reimbursement/pricing, and are intended to assess clinical effectiveness, safety, and cost. Four additional domains are further considered in what is called a “full HTA”: ethical, legal, social, and organizational aspects. The ethical aspects have long been the subject of debate regarding how they should be processed. It would be important if the following questions could be answered: Who publishes full HTA reports and how? Which methods are used in the ethics domain? What kind of results do they produce? However, such a “mapping of the field” turns out to be difficult. Despite the existence of international HTA registers, we were not able to compile a comprehensive sample of full HTA reports. Therefore, the aim of our study was rather to explore a) substantially: Which information can be expected to be (easily) found, which can only be obtained with considerable effort, and which remain (for the time being) in the dark? And b) methodologically: Is it possible to do meaningful meta-research in this field?

**Methods and results:**

In the attempt to explore the possibilities of meta-research, we were able to track down and analyze thirty-nine full HTA reports from six countries.

**Conclusions:**

While not representative of the whole field, this analysis shows the possibilities and challenges to meta-research, but nonetheless also provides some substantial insight into the characteristics of such reports, with a particular focus on the methods used to process ethical aspects.

## Background

Health technology assessment (HTA) is the “systematic and multidisciplinary evaluation of the properties of health technologies and interventions covering both their direct and indirect consequences” (1;2). As a tool of scientific political advice, HTA has a distinct role in determining the added value of a given technology over and above existing ones (3). Therefore, HTA reports are more specifically written for decision makers in the healthcare system, particularly regarding questions of reimbursement (in nations with social health insurance) and/or pricing and, at least, *intended* to cover more than just *clinical effectiveness* and *safety* (4;5). In addition to the aforementioned and *cost and economic evaluation*, four other nonclinical assessment domains of a technology are also considered in what is called a “full HTA” (e.g., (5–9)): *ethical*, *legal*, *social*, and *organizational* (ELSO) aspects; social aspects are sometimes also denoted as *patient* aspects.

However, these nonclinical domains, especially the ethical, legal, and social domains, are processed less frequently (52–65 percent) than the clinical (100 percent) and economic aspects (>85 percent) (9;10). The focus on clinical aspects may be plausible for HTA that are restricted to pure drug assessments (11), as the ethically sensitive information in this context is usually already covered by the clinical efficacy, safety, and economic evaluation. However, the reasons for excluding particularly the ethics domain are not limited to reasons that are inherent to the technology itself. They, thus, also occur in HTA on nonpharmacological interventions. Furthermore, the danger is also expressed that the consideration of ethical aspects could lead to a bias in HTA because it would lead to decisions about a technology ultimately no longer being made on the basis of objective evidence (= clinical and economic data) presented as unbiased as possible, but on the basis of soft data (= *ELSO*), non-scientific or “political” and, thus, less objective reasons (12).

The little attention paid to ethical aspects may be appropriate in individual cases, as the decision to include and weight the individual domains can vary depending on the technology, the healthcare system or other contextual factors (4;13). Therefore, some people argue that the processing of ethical aspects is only necessary for “controversial technologies” (13). There are other reasons given in the literature for covering the ethics domain insufficiently or not at all, for example, a lack of competence in the HTA team, the absence of standardized methods, or a shortage of time and resources (e.g., (12;14)). However, a survey among 28 European states concluded that ethical analyses are “indeed practiced by a large majority of countries/institutions” (4).

From an international perspective, “ethics” is perhaps the most interesting of the ELSO domains, as there are well-established international frameworks in bioethics, public health- and research ethics (15–17). However, ethical aspects are often seen as intertwined with legal, social, and organizational aspects (2;18). Also, there are currently no uniform methodological standards for the systematic consideration of ethical and, for example, social aspects in HTA. Despite the absence of a unified understanding of ethics as a HTA domain or of ethical analysis as part of HTA, the ethics domain can be conceptualized as a pervasive domain that can normatively assesses aspects from all domains (19). While there are explicit and well-developed approaches (20–23), methodological ambiguities can lead to poor reporting. Furthermore, ongoing discussions within the field of bioethics addressing the absence of established methodological standards (24) could potentially lead to uncertainty among authors. Therefore, it is important to investigate how the ethics domain is set up in an HTA report and whether and how, for example, the methods used were reported (25;26).

A better understanding of how the ethics domains are methodically processed is all the more important when it is considered that HTA reports are intended to provide information for healthcare decisions. Although evidence from research is only one of the many factors considered in policy development, there is an increasing recognition of its potential value (27). As both HTA and policy making are inherently value-driven processes, there should be transparency about the evidence and its validity and reliability. There are various initiatives to expand and facilitate international cooperation in HTA, such as the Core Model® by EUnetHTA. The EU regulation, which will be applicable from 1 January 2025, even stipulates a centralized assessment at European level. However, this only applies to the clinical aspects; the nonclinical aspects will continue to be dealt with at national level (28). Furthermore, the fact that 31 percent of the HTA agencies in Europe allowed reports in English in addition to the respective national language shows that international orientation is also envisaged at the level of national agencies (4). There are also registers for international searches for HTA reports, such as the International HTA database (29;30).

Against this background, it is surprising that the report “Mapping of HTA national organizations, programmes and processes in EU and Norway” (9) does not shed light on the publication and dissemination of reports. This would be particularly interesting in the case of *full* HTAs, since these, in contrast to mere drug assessments, are not necessarily compulsory within the respective countries, for example, regarding decisions on what should be covered by the health insurance fund. Thus, there are a number of important questions left open, such as, Who publishes full HTA and who reads it? How are full HTA reports reported internationally? Which methods are used in the ethics domain? What kind of results do they produce, and (how) are these integrated into the overall report?

The field of meta-research on HTA is still in its nascent stages, and it is not feasible to address all relevant questions within the confines of a single project. The Supplementary Material of this manuscript contains anecdotal insights into current challenges for meta-research regarding full HTA reports (see Supplementary Material, Appendix 1 and Discussion section). Before the above questions can be addressed in detail, it must first be a) substantially clarified which information can be expected to be (easily) found, which can only be obtained with considerable effort, and which remain (for the time being) in the dark; and b) methodologically, whether meta-research on full HTA is possible in principle and what kind of quantitative and qualitative results are realistic with such an approach. The aim of the research project was to conduct a pilot meta-research study on full HTA in order to gain a profound understanding for critical self-reflection. In this context, the present study enables preliminary insights into full HTA and especially its ethics domains.

## Method

### Identification of HTA agencies and HTA reports

Firstly, we developed a search strategy to identify those HTA agencies that publish full HTA. One author (HK) searched for specific programs for full HTA reports on fifty-seven agencies web sites from thirty-six countries (membership list of the INAHTA network, see Supplementary Material, Appendix 2). The search was conducted in October 2022 without restriction on the publication period; an update was subsequently implemented in February 2023. The identification of potentially relevant agencies and reports was finalized by manually searching further agencies via Google (February 2023). In addition, we screened the publications of agencies and the international HTA database (29;30) using appropriate filtering functions. The screening was conducted by the authors in English (HK), German (HK), French (MM), and Portuguese (CF). The eligibility criteria were agencies where we could assign at least one “full” HTA report or a report that includes an “ethics domain,” and these reports were accessible in full text in one of the four aforementioned languages. A small method pilot was conducted to compensate for the lack of our language skills. Two reports each from Norway and Sweden were translated with the help of the AI-based translation software deepL.com to English (on 5 May and 15 June 2023).

If an agency did not explicitly list any full HTA programs on its web site and no full HTA report could be identified via search filters on the agency’s publications site, the following procedure was followed: if there were no hits or more than 30 nonspecific hits, the agencies were contacted and asked to send us a list of the corresponding full HTA reports (January and February 2023). Thirteen agencies were contacted, and five responded. Of these, one agency provided a total of eight reports, six of which were included in the final analysis.

If fewer than ten reports were identified, all were included in the analysis. If there were more, we selected a sample of ten full HTA reports. In order to increase diversity, we applied soft criteria for the selection: We first included ten reports from an agency in chronological order, that is, how they appeared in the respective list of the agency. We then checked whether reports were written by the same t groups of authors or were published in the same years. If this was the case, some of the reports were sorted out and replaced by other reports from the respective list, again in chronological order. If a decision had to be made between two or more reports, topics apparently particularly suitable for ethical considerations were favored.

### Analysis of the HTA reports

The matrix for the data extraction of the HTA reports included was developed involving all four authors; a first draft of the matrix was developed mainly by HK, with CF and MM critically revising. Several items (categories) of the matrix were deductively determined based on previous experience with HTA reports. There were items that only asked for their presence in the report (yes/no answer categories, e.g., whether there was a declaration of possible conflicts of interest), items that asked for a numerical value (e.g., number of pages) and those that needed more qualitative information using subcategories (e.g., which methods were used). The matrix was then pilot tested with selected HTA reports independently by all authors at different stages and discussed in detail several times in the group. If necessary, decision rules were defined or the items were further specified (e.g., what counts when counting the pages). The matrix was inductively expanded as it progressed.

In the final analysis matrix, ten items pertain to the agency web site and twenty-seven to the report. In addition, there were twenty-four items specific to the ethics domain and the methodology used. For the analysis, the table of contents, introduction, overall conclusion of the HTA reports, as well as the methods and results section of the ethics domains were carefully reviewed. Only explicit indications of the outcome categories (e.g., “issues” or “conflicts”) were considered for inclusion in the analysis. Such outcomes were quantified only when they were easily countable (e.g., by subheadings, bullet points, or tabular presentations). By doing so, interpretative evaluations, such as the identification of arguments in a text passage, were avoided. This analysis was primarily performed by one author (AS). As the analysis of the ethics domain is more interpretative, however, these 24 items, as well as the assessment of the user-friendliness of the web site, were checked 100 percent by another person (HK) in the original documents (Cohen’s kappa 0.90). The accuracy of the descriptive items (e.g., number of pages) was confirmed by another person for every tenth report. The information extracted was then synthesized to identify quantitative and qualitative differences not only in between agencies but also within an agency.

### Explanations of specific approaches used for the ethics domain

We differentiated the following approaches regarding the methods used in the ethics domain:

*Literature review*: There are a variety of approaches to ethics literature reviews that can be more or less a) systematic and b) critical (31). Ethical issues are not always very specifically addressed in the literature; at the same time, ethical aspects can be transferred well through critical reflection. As a result, the searches for ethics literature are not always specific.

*Interaction with external experts*: In addition, experts who are not members of the author group may also be involved. This especially includes potential users of the HTA report and people (professional or lay) involved in the care of an affected person (further elaborated in Table 3).

*Theory-based reflection*: Authors reflect on whether (further) ethical aspects need to be considered based on specified theoretical approaches for dealing with the ethics domain and, as a rule, against the background of experience with ethics topics and knowledge of relevant literature. This critical reflection can take place by oneself or in a group.

## Findings

### Who publishes full HTA reports in English, French, German, or Portuguese?

There were 39 HTA reports from seven national HTA agencies that were finally included in the analysis (see Supplementary Material, Appendix 3): Canada’s Drug and Health Technology Agency (*n* = 4), the Swiss Federal Office of Public Health (*n* = 10), the Institute for Quality and Efficiency in Health Care (*n* = 10), the Ludwig Boltzmann Institute for HAT and the Austrian Institute for HTA (*n* = 5), the Swedish Agency of HAT and Assessment of Social Service (*n* = 8), and the Norwegian Institute of Public Health (*n* = 2) (see Table 1). This sample represents only a (presumably) small part of the activities at full HTA. It was not possible to determine a full sample of full HTA or the agencies that publish such reports with our approach. With the exception of Sweden, all reports were published between 2016 and 2023, with a maximum range of 5 years within an agency. For Sweden, a report from 2008 was included and the time span was over 13 years (see Supplementary Material, Appendix 3).

**Table 1.** Basic characteristics of the reports from the seven HTA agencies (in six countries)

	Reports analyzed (total of “full HTA” reports)	Funding of reports (as reported in the main document)	Author groups of the reports (as reported in the main document)	No. of authors
Canada	4 (of 4)	Public funding: 100%	Mixed group: 25%	2–14
Switzerland	8 (of unknown)	—	External authors: 100%	5–12
Germany	10 (of 21)	—	External authors: 100%	4–12
Austria	5 (of 5)	—	Mixed group: 20% External authors: 40%	2–4
Sweden	8 (of unknown)	Public funding: 13%	External authors: 13%	5–29
Norway	2 (of 2)	—	—	5–8

HTA, health technology assessment.

Only one agency provided information *within the report* about the funding and 64 percent of the reports contained information on conflicts of interest (see Table 2). There were differences in the composition of the author groups: While some agencies trusted exclusively on external author groups, others were authored by mixed groups (members of the agency and external authors, Table 1). At the same time, only one agency stated explicitly which authors processed which domains in half of the reports.

**Table 2.** Main characteristics of the thirty-nine full HTA reports

	Main report (pp. = pages)	Summary(within the main document)	Appendix included/excluded (from the main document)	Protocol available/amount registered	CoI statement (reported in the main document)	Lay version	Scientific publication	Other documents(incl. “main reports” in other languages)
Canada	English 100% 16–218 pp., mean 111	English 75% 6–7 pp.	100/0% 13–244 pp.	75/100%	50%	25% 2 pp.	0%	2× **Short form** English: 2 pp.
Switzerland	English 100% 44–221 pp., mean: 110	English 90% 2–4 pp.	90/10% 20–133 pp./ 205 pp.	80/0%	20%	0%	0%	2× **Main report** German: 89 pp. 6× **Short form** German: 12–89 pp. 7× **Feedback** ^a^ English: 8–49 pp. and German: 15–21 pp. 3× **Feedback** ^a^ French: 8–32 pp.
Germany	German 100% 38–113 pp., mean: 55	German 100% 3–10 pp.	100/0% 129–268 pp.	100/40%	100%	100% 3 pp.	0%	3× **Main report** English: 54–100 pp. 10× **Feedback** German: 1–36 pp. 8× **Provisional reports** German: 36–100 pp.
Austria	English 100% 35–109 pp., mean: 85	German 100% 2 pp. English 100% 2 pp.	100/0% 6–203 pp.	0%	100%	0%	20%	—
Sweden	Swedish 63% 45–109 pp., mean: 79 English 38% 27–432 pp., mean: 191	Swedish 50% 3–5 pp. English 25% 1–19 pp.	50/50% 4–121 pp./ 11–121 pp.	50/100%	50%	13%	63%	6× **Short form** English: 2–22 pp. 1× **Statement of ext. auditors**: 10 pp. 1× **External appendix** English: 151 pp.
Norway	Norwegian 100% 38–94 pp., mean: 66	Norwegian 100% 4–9 pp.	100/0% 25–67 pp.	50/ 0%	100%	0%	0%	—
Mean or most popular (all 39 reports)	English: 56%, 89 pp.	English: 69% (including “other documents”)	100%	64/28%	64%	30%	15%	

a The stakeholder feedback in different languages was published partly together and partly in linguistically separate documents.

HTA, health technology assessment.

Three criteria were used for evaluating the web site usability of the agencies: 1) navigation in English, 2) access the main report via “1 mouse click” from the reports webpage or (if not existent) the main agency web page, and 3) clear title for the documents. Despite this approach was rather simple, it can be noted that differences were apparent. Three agencies published the reports in a “user-friendly manner.” In a few cases (8 percent), ambiguities were found in the description of the types of the documents (e.g., full or short report, laymen version). In one instance, navigation was not possible in English, which may be disadvantageous for international findability.

All agencies assessed the five nonclinical aspects of the technology according to the Core Model® at least in one HTA report (economic and ELSO aspects, Supplementary Material, Appendix 4). In addition, four other domains were mentioned: a) environmental (Canada); b) decision making, communication, patients, privacy, consent, and patient involvement (Austria); c) ethnicity and culture (Sweden); and d) others (Switzerland and Sweden).

### How are full HTA report reported internationally?

The reports averaged eighty-nine pages, but the range in our sample varied greatly across the agencies (Table 2). Some of the reports were, on average, well below 100 pages (Germany mean 55 pp., Austria 85 pp., and Norway 66 pp.) and the others above (Canada 111 pp. and Switzerland 110 pp.). The HTA reports in English from Sweden were more than twice as long as those in Swedish (191 vs. 75 pp., respectively, no qualitative assessment). The reports in two countries (Switzerland and Austria) were primarily published in English, although this is not the national language. In addition, the length of the reports also varied greatly within the countries. In many cases, there were differences of more than 100 pages (correlations with the respective technology were not investigated but can be assumed). Summaries or short forms in English were found for 69 percent of the reports (nineteen within the main document and eight in a separate one). One (non-English-speaking) agency did not publish any document in English and two published English information for all reports (at least, in a short form). All agencies provided Supplementary Material. The size varied widely (13–244 pp.), and the Supplementary Material was predominantly found in the same document as the main report (see Table 2). Most agencies (64 percent) also published protocols to the reports, but these were only partially preregistered in appropriate registers (total 28 percent of all reports). Only one agency consistently published laymen versions of its reports and two others did so for some of them (total 30 percent). In two agencies, scientific publications could be identified for 15 percent of the reports. Some agencies provided other documents (e.g., protocols, annexes, short forms, scoping reviews, provisional reports, or stakeholder feedback) in addition to the main report.

### Which methods are used in the ethics domain?

Three agencies addressed ethical aspects consistently in a separate domain and three agencies mixed separate and shared domains for ethics. The length of the ethics domains varied from 0.5 to 65 pages, (mean 13.3 pp.) (Table 4, 1.1). The methods were described compactly on 0.5 to a maximum of four pages (mean 1.4 pp., 2.1); every report had such a description. All HTA reports stated that they used a *literature review* for the ethics domain (2.2), with 74 percent conducting their own specific search and 26 percent using results from the general literature search within the HTA project. The labels used for this vary between “systematic review,” “oriented search,” and simply “review” (Table 4, 2.2). The number of hits found in these literature reviews varied from 0 to 149 hits (mean twenty-eight hits). The second possible source of information relevant to the processing of an ethics domain was an *interaction with external experts.* This strategy was only regularly chosen by one agency and in some reports from other agencies from our sample (total 30 percent) (Table 4, 2.3). Only three reports (from two agencies) stated explicitly whether a qualitative or quantitative approach was chosen for the interaction. A qualitative approach was chosen in two cases and a quantitative approach in one case. The method most frequently used was individual interviews, followed by group discussions. The largest group of experts consulted was patients (Table 3), where up to sixty-five individuals interacted (Table 4, 2.3). *Theory-based reflection* was the third strategy, which again was quite common among all agencies (total 64 percent).

**Table 3.**
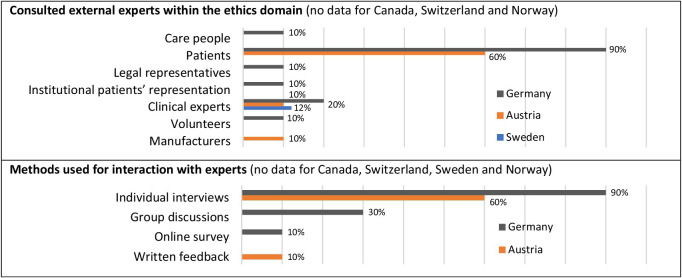
Interaction with experts within the ethics domain: who and how? (also see Table 5, 2)

**Table 4.** Characteristics of the ethics domain

	1. Domain	2. Methods						3. Results		
	Ethics in separate/shared domain	2.1 Reporting on methods	2.2 Literature review((non-)SR = (Non-) systematic review, OS = Oriented Search, R = Review)	2.3 Interaction with external experts(see Supplementary Material, Appendix 5)	2.4 Theory-based reflection	2.5 Theoretical background(see Supplementary Material, Appendix 5)	2.6 Reporting on Synthesis	3.1 Reporting on Results	3.2 Presentation of the results(narrative/Tabular)	3.3 Ethics in overall summary
Canada	100/0% 0.5–23 pp.	100% 0.5–4 pp.	SR: 100% 11–149 pp., mean: 72	all reports: no info	75% 25%: no info	Deontological 25% Principlism 50% 25%: no info	50%	100% 1–15 pp.	nar: 100% tab: 100%	100%
Switzerland	0/100% 1–15 pp.	100% 1–3 pp.	SR: 40% Non-SR: 60% 5–22 pp., mean: 8	all reports: no info	60% 40%: no info	Principlism 30% Socratic 20% Integrate HTA 10% EUnetHTA 10% 70%: no info	all reports: no info	100% 0.5–9.5 pp.	nar: 100% tab: 40%	50%
Germany	90/10% 6.5–65 pp.	100% 1 p.	OS: 100% 4 pp.	90% 2–15 pp. 2–5 people	100%	Principlism 50% Socratic 90% Integrate HTA 10%	90%	100% 1.5–9,5 pp.	nar: 100% tab: 90%	90%
Austria	80/20% 2–23 pp.	100% 0.5–4 pp.	SR: 60% Non-SR: 40% 34–80 pp., mean: 8	60% 6 pp. 6 people 40%: no info	80% 20%: no info	Principlism 20% Socratic 20% EUnetHTA 20% 70%: no info	all reports: no info	100% 2–13 pp.	nar: 100% tab: 80%	80%
Sweden^a^	63/25% 5–20 pp.	38% 1 p.	SR: 25% Non-SR: 25% 0–50 pp., mean: 8	13% 10 pp. 10 people 87%: no info	25% 75%: no info	SBU Guidelines 25% 75%: no info	all reports: no info	83% 0.5–15 pp.	nar: 75% tab: 0%	50%
Norway^a^	100/0% 2.5–35 pp.	100% 0.5–1 p.	R: 50% 22 pp.	50% 50%: no info	50% 50%: no info	Norwegian Knowledge for Health Service 50% 50%: no info	50%	100% 2–35 pp.	nar: 100% tab: 0%	50%
Mean (all 39 reports)	13.3 pp.	1.4 pp.	85%	36%	64%	Socrat. 33%, Principl. 26%	30%	5.7 pp.	nar: 100%, tab: 54%	72%

a Since translation programmes with AI were (partially) used for the assessment, “no information” or “not found” means rather that it remains “unclear” whether information was provided in up to 100% of the Norwegian and 25% of the Swedish reports.

The theoretical backgrounds used were deontological and Socratic approaches, especially Hofmann’s question catalogue (20) and principlism (15;33). Supplementary Material, Appendix 5 provides a more detailed overview of the approaches used and explains them. Only one agency provided a description of the synthesis of the (main) findings of the ethical analysis, at least in the sense of a thematic condensation or summary, in most of its reports (88 percent); in total 30 percent did so (Table 4, 2.6).

### What are the results of the ethics domain and how are these integrated into the overall report?

Half of the reports assessed published “ethical issues.” Specific “concerns,” “aspects,” “values,” “judgment criteria,” and “questions” were the outcome dimensions once each. The number of results varied, sometimes a few (three to six concerns, questions, or conflicts), sometimes many (up to fifty and fifty-three issues and aspects) (Table 5). The results were presented narratively in all reports. About half of the reports also relied on a (supplementary) tabular presentation (Table 4, 3.2). The results of the ethics domain were taken up in the overall results (main conclusion) in most (72 percent) reports in a comprehensible way. Results of the ethics domain were not mentioned in the main conclusion in 50 percent of the reports from Switzerland, Sweden and Norway (Table 4, 3.3).

**Table 5.** Types/categories of results from the ethics domain (multiple responses possible)

	Issues	Aspects	Concerns	Questions	Conflict	Challenges	Values	Judgment criteria	Considerations
Canada	75% *n* = 10–50		25% *n* = 3	25% *n* = 6					25% *n* = 6
Switzerland	80% *n* = 0–3			10% *n* = 9	10% *n* = 4	10% *n* = 4			
Germany		100% *n* = 4–33		10% *n* = 13				10% *n* = 7	
Austria	20% n.s.	40% *n* = 53				20% *n* = 63			20% n.s.
Sweden	12.5% *n* = 16	50% *n* = 2–6			12.5% n.s.		12.5% n.s.		
Norway		50% *n* = 2		50% *n* = 3					

n.s., not specified.

## Discussion

Our attempt to map the field of full HTAs internationally focused on not only who publishes these reports and how but also methods that are used in and results of the ethics domain. These are questions that, in principle, can be addressed by meta-research, which is still scarce in this area. However, an observation of our attempt is that the material that can be identified and collected with reasonable effort is not sufficient to fully answer the interesting questions. As we describe in detail in the Supplementary Material, we adjusted our initial research question twice because we did not find viable search options (e.g., filters) for our purposes. All sorts of initiatives and measures to increase international findability, especially through a more consistent use of (English) terms, are very welcome.

Nevertheless, in order to make some progress, quantitative and qualitative findings are described. As the sample is too small to be representative, the quantitative findings do not provide a comprehensive overview but exemplary differences in the current status of full HTA reports. This does not impinge upon the qualitative aspects of this work that provide some pointers for the further development of HTA and particularly the ethics domains. The focus here is on the presentation of differences and variations (where representativeness is not a quality criterion).

### Who publishes full HTA reports?

The identification of a comprehensive sample already posed unsolvable challenges (see Supplementary Material, Appendix 1, cf. also (26)) that shaped the final study design. This means that despite a systematic approach and thorough screening, including contacting agencies, we were probably not able to identify all publishing agencies or retrieve a full sample of full HTAs. The primary causes of this limitation was poor international searchability, inconsistent understanding of full HTA, and probably also our focus on four languages (which is why we only identified six reports through direct enquiries). This is unsatisfactory given that such meta-research provides an important basis for improving future (full) HTA.

Against this background, thirty-nine full HTA reports from six countries and seven agencies were analyzed. For three agencies, all identified reports were analyzed (full sample). For two agencies, approximately half of the identified reports were included in the analysis. For one agency, the total number of full HTAs was unclear. The respective HTA agencies generally function as editors of the reports. In addition, some agencies used their internal methodological expertise to co-write reports in mixed author groups, while others commented explicitly on the reports written by an external author group or published an “external report” with no clear editorial involvement. Integrating internal staff can bring continuity to the writing process (quality assurance) and enables less experienced author groups to participate (nonexclusivity). At the same time, this requires more staff capacity. Some agencies in the European region do not provide any staff, while others have a staff capacity of up to 670 people (4, S. 28). The number of authors of the HTA reports in our sample varied between two and twenty-nine. We see a wide range within many agencies, which could be explained by less extensive topics (and, therefore, be appropriate). However, full HTAs are very comprehensive, require a number of specific methodological skills, and thrive on an interdisciplinary perspective. Therefore, it could be questioned critically whether a small group of authors can fulfill all these requirements. In this context, it would also be interesting to know which authors have worked on which parts in detail, and who is responsible for the domain. Only 5 percent (*n* = 2) provided this information in our sample, although full disclosure would be desirable (transparency). Reports are written in an interdisciplinary way, bringing together professions with their own cultures, languages, and traditions. The different perspectives and complex interrelationships can also lead to misunderstandings (19). In order to enable a public corrective, HTA reports should be well-structured, comprehensive, and easily accessible.

We further identified structural shortcomings in the user-friendliness of the accessibility of the reports. Even though our approach to evaluation was rather quick and dirty, this should be seen as an indication of the potentials that lie in a user-friendly interface. From an international perspective, the possibility to navigate and identify HTA reports in English is key. About every third agency in Europe already produces main reports in English in addition to the national languages (4).

### How are full HTA reports reported internationally?

In addition to the five nonclinical domains from the Core Model®, we have identified further domains. One could also discuss whether environmental issues should cover on a regular base. The difficulties of demarcating the ethics and social domains are well-documented (19). Therefore, new domains should only be introduced with explicit demarcation to existing domains. From an international perspective, referring to uniform domain descriptions is desirable as it facilitates the accessibility, comparability, and adoption of results. It also allows proposing or defining methods suitable for the processing of a specific domain.

Protocols were published for about half of the reports (46 percent). Some agencies published protocols on the specific web site of the report, while others (28 percent) relied on a publication in a registry (e.g., PROSPERO). Central registries greatly facilitate international searches, increasing the probability of receiving early information about upcoming and ongoing projects.

The financial background (13 percent addressed funding and 64 percent conflicts of interest) was not reported in a consistent and standardized manner. It has become standard practice for scientific results published in journals to include this information directly in the manuscript—a practice that could also be expected to be followed for HTA reports. However, it could be argued that if the funding is not explicitly mentioned, it can be assumed that the report was financed by the agency itself. Still, there is currently no transparency or clear standard regarding the reporting of such information.

The question of how results should be published and disseminated depends on the target audience to be reached. However, the question of who reads and uses full HTA reports remains unanswered. Although HTA reports are often assigned identification numbers by the agencies, these were not sufficiently unique to permit the tracking of their subsequent use and application. In contrast, study registration numbers are sufficiently specific to facilitate the follow-up of related studies (see IntoValue (bihealth.org) (32)). In addition to scientific journals, databases, and registers, the wider interested public could also be informed, for example, through press releases. A straightforward online search (Google) in English yielded the following result: Only one agency regularly published press releases, which were also reflected in a press echo. In one country, some reports were picked up in the press without there being a press release.

### Which methods are used in the ethics domain, what are the results of the ethics domain, and (how) are these integrated into the overall report?

It should be noted at the outset that not all information that we sought and deemed potentially relevant was found in the reports. Although this reduces the validity of some (more general) statements about full HTA, it still allows important qualitative differences to be described.

In our sample, the main approach to information gathering in the ethics domain was a form of literature review, followed by theory-based reflection and, in a few cases, supplemented by interaction with stakeholders/experts. The fact that the latter was quite rare could be explained by the limited budget and rather elaborate methods that require additional qualifications. However, stakeholder engagement in HTA should be “politically mandated and institutionalized” (4). This includes the definition of patient-relevant outcomes for the clinical domains and the stakeholder perspectives on ELSO aspects. There seems to be certain awareness, because the majority (64 percent) of the agencies that had not yet involved stakeholders in 2005 and 2010 planned to do so in the future (34). Even if these statements do not refer to certain domains, they provide good arguments to develop proper methods for stakeholder engagement in nonclinical domains.

There was a wide variation in the types of categories of results targeted by the ethics domain when not only comparing the national HTA agencies but also within the reports of an agency: from abstract topics and values to concrete assessment criteria and questions. The rather vague term “(ethical) issue” dominated (see Table 5). However, the respective categories were seldom adequately defined. It is not clear whether, for example, “issue” and “challenges” mean the same thing or something else. Without such clarifications and (more) agreement on the categories, the ethics domain also remains somewhat diffuse. So, it could be discussed whether an international understanding about the categories is desirable and, if so, what measures should be taken to work toward this. Issues or aspects tend to state what is ethically problematic, for example, while criteria are intended to orient one’s own decision making; questions, on the other hand, tend to stimulate reflection (inviting one to make certain considerations about a technology with which one is grappling). Thus, an ethics domain may serve different goals or functions that are not always entirely comparable and may require different methods. Differences in the number of issues, aspects, concerns, criteria, and so forth reported are probably due to the specifics of the technology and need to be assessed in the light of these (which we did not do). Yet, the method used to analyze the literature and different ways of summarizing or condensing the (intermediate) results could also play a role here. Agencies should define their expectations from the authors, which may include, for example, a descriptive presentation of ethically relevant aspects or a normative evaluation of a technology; a comprehensive and detailed—and thus probably rather long—list of issues and aspects, or a concise and focused set of concerns, questions, or conflicts. It also seems unclear whether and how the results of the ethics domain should be merged with the main results of the report (e.g., as recommendations—provided an HTA agency has the mandate to include recommendations in its HTA reports).

Only explicit information was considered in determining the theoretical background. The HTA reports that did not made the theoretical background explicit can by no means be described as “theory-free,” however. We encountered approaches, for example, which we would describe as “eclectic,” but have not coded it because it was not explicitly described by the authors themselves. Finally, while synthesis methods are not yet as sophisticated as the synthesis of descriptive statistical information, there is some work that can provide good guidance in this context: (35–37).

## Limitations

We evaluated the *reported* information identified on the web sites and all accessible files. In this context, “not reported” should not be equated with “not available/not done.” At the same time, certain result categories are involve interpretative tasks. In order to guarantee the reliability of the assessment, these interpretative categories were evaluated by two independent raters, based on good examples of the fulfillment of a characteristic (e.g., reporting whether theory-based reflection has taken place as part of the ethical analysis). Surveys are carried out to supplement missing information or correct inaccuracies in more comprehensive studies, such as the EU’s mapping-the-field report. This was not practicable in the context of this study. It is also quite possible that our results on scientific publications underestimates the results actually published in (medical) journals; but these activities are somehow “invisible” until they are (more directly) linked to the agency or specific HTA reports. It is unfortunate that, due to the lack of tracking options, it remains often unclear what impact the reports have after publication. So we report on a relatively small sample of seven national HTA agencies. We assume that there are many more agencies that publish full HTA reports that we were not able to identify due to not only language barriers but also difficulties in finding such HTA reports on the respective web sites or the INAHTA database. However, we see this limitation primarily as an important finding of our study, namely, that it is currently difficult to identify full HTA reports—which significantly reduces the value of such reports in the international arena.

Our results describe primarily qualitative differences of the ethics domains; however, it should also be noted that “newer” and “older” HTA reports (range within agency: 2–5 yr; except SWE with 13 yr) were taken into account which might *in part* explain some of the differences. Some descriptions of the methods and results were much elaborated. They offered a detailed step-by-step description, elaborate on critical or challenging aspects, and embed the results in the main conclusion. However, these are impressions that were not gathered systematically. We would, thus, welcome more in-depth qualitative analysis of the reporting. Such empirical information could be valuable for the development of a best practice of approaching and reporting ethical issues in a HTA report.

Although searches were conducted in four languages, there are language limitations to the study. If reports cannot be searched in English (or another language that “happens” to be spoken by the researchers), the question arised regarding whether researchers should use automated translation programs. We tried this for two agencies and were, thereby, able to include more reports in the analysis. The quality of the translation cannot be appraised by the researchers. One solution could be that the agencies validate any result that is based on an automated translation. As such a validation process was not carried out in the context of our study, the corresponding results are marked “uncertain” in the tables.

It was not possible to classify the reports thematically because of the small sample size. The relevance of certain domains depends strongly on the respective technology. Therefore, a narrower subgroup analysis would probable bring further important quantitative and qualitative findings. Moreover, it is unfortunately not possible to make statements about changes within agencies over time. On the one hand, the samples per agency are often too small and, on the other hand, the variability observed is highly dependent on the group of authors and/or respective technology. Thus, possible policy changes within agencies toward the publication strategy of HTAs and the handling of ethics domains could not be ascertained in our data.

## Conclusions

It is to be expected that full HTAs are, or will remain, less widespread than systematic assessments of the clinical effectiveness and economic aspects of a technology. Nevertheless, a number of such reports exist internationally. Our attempt to map the field of full HTA with a focus on the ethics domain has shown the difficulties that meta-research faces in this area. It also highlighted the strengths and weaknesses of various strategies (searching specific databases, via HTA agencies, enquiries to HTA agencies, etc.) to obtain full HTA reports in the first place. Nevertheless, it also positively demonstrated what information can be extracted, analyzed, and assessed about, in and from HTA reports. Meta-research is therefore possible, even if the preconditions for this could be better.

Our study identified three areas for action: 1) To improve searchability, existing classifications of domains, such as those found in the Core Model®, or the term “full HTA,” should be used more clearly in order to identify such reports more easily. 2) Differences in the structure and treatment of nonclinical domains should especially be addressed and ideally linked to a best practice approach. Although methodological differences in the ethical analysis are justified, also due to contextual factors of the respective countries, a better comparability based on similar structure and terminology (e.g., “issues,” “challenges,” etc., and whether this results in different methods) would be desirable. 3) In addition, the quality of reporting is, at least in our sample, often unsatisfactory. The trends described here should be confirmed or corrected by a larger sample to provide additional empirical evidence for the further development of the international full HTA landscape. Our findings support the conclusions of the mapping review in the EU and Norway, which call for constructive discussion on “methodologies that will fit to cross-border cooperation” (4, p. 9).

To facilitate better meta-research on full HTAs, *HTA agencies* (editors) should:

- consistently label reports that assess more than just efficacy, safety, and/or economics, that is, that also address ELSO aspects, as full HTAs. This terminology should appear in the title of each report, but also in program titles/funding lines.
- give reports identification numbers (IDs) that make them internationally searchable via internet searches.
  Consider to also publish an English version (short or long version).

To increase the (international) usability of full HTAs, *HTA agencies* (editors) should:

- engage with *Decision makers* and c*ritically reflect* how full HTAs actually inform their decision making. Evidence-based policy making is not without challenges; however, multiple guidelines address them and offer orientation, for example, the World Health Organization guide for evidence-informed decision making (38). They should provide feedback whether they are satisfied with how nonclinical domains are covered.
- *develop international standards* on full HTAs for *authors* and *editors.* These should include methodological specifications, objectives, and guidelines for approaching and reporting of ELSO aspects. Even or especially in the case of a certain (justified) methodological diversity in the field, reporting standards that facilitate international accesses are indicated. Furthermore, authors should be encouraged to e*xplain and justify* the choice of method to the reader (comprehensive reporting). As long as specific international reporting standards for ethics domains in HTA are missing, different approaches on (systematic) literature reviews in ethics are presented in, for example, (35;37;39); the RESERVE guideline offers twenty-two items for reporting on systematic literature reviews of ethical literature.

## Supporting information

Kahrass et al. supplementary material Kahrass et al. supplementary material

## Data Availability

https://doi.org/10.6084/m9.figshare.24771948.v4. Appendix 1–5: (figshare.com) DOI: 10.6084/m9.figshare.24771948.v4 A list of all 39 analyzed “full HTA” can be found in Appendix 3.

## Supplementary material

The supplementary material for this article can be found at http://doi.org/10.1017/S026646232400480X.
