# Supplementary material for: Ethics domains in full health technology assessment reports: an attempt to begin mapping the field
Source: Int J Technol Assess Health Care. 2025 Apr 8;41(1):e22. doi: 10.1017/S026646232400480X (PMC12019511; doi:10.1017/S026646232400480X)
Supplement: Kahrass et al. supplementary material [file S026646232400480Xsup001.docx]

**Ethics domains in full health technology assessment reports: an attempt to begin mapping the field**

**Appendix for the Manuscript**

Appendix 1

**Background on the development of the project**: As writers of HTA reports, we were interested in the question who finally reads these reports. After several discussions, we came to the conclusion that we neither know the specific kind nor the number of recipients and are not able to survey them. Moreover, reading leaves no traces and the interests and conclusions of individuals in and from the results remain unknown. To continue, we reformulated our research question and asked: Where, by whom, and for what purpose are published HTA reports cited (qualitative and quantitative analysis)? This question was oriented by studies that did a follow-up of published randomized controlled trials (RCT). In contrast to HTA, study registrations for RCTs are more common, whereby registration numbers exist by which the studies can be identified in a common internet search [see 1, 2]. After thorough piloting, we find that tracking of certain HTA reports is not possible, as there are not sufficiently unique ID codes, and the titles are often not sufficiently specific. The latter leads to an unmanageable number of hits of scientific articles with similar or same questions.

References:

1. Riedel, N., Wieschowski, S., Bruckner, T., Holst, M. R., Kahrass, H., Nury, E., Meerpohl, J. J., Salholz-Hillel, M., & Strech, D. (2022). **Results dissemination from completed clinical trials conducted at German university medical centers remained delayed and incomplete. The 2014-2017 cohort.**Journal of Clinical Epidemiology, 144, 1–7. [https://doi.org/10.1016/j.jclinepi.2021.12.012](https://www.sciencedirect.com/science/article/pii/S0895435621004145?via%3Dihub)
2. Strech D, Sievers S, Märschenz S, Riedel N, Wieschowski S, Meerpohl J, Langhof H, Müller-Ohlraun S, Dirnagl U (2018). **Tracking the timely dissemination of clinical studies. Characteristics and impact of 10 tracking variables.**[F1000 Research; 2018 Nov 29;7:1863](https://f1000research.com/articles/7-1863)

Appendix 2

| **Austria (AIHTA , GOeG, LBI-HTA)** | Argentina (IECS) | Belgium (KCE) | Brazil (ANS, CONITEC) | **Canada (CADTH, OH, INESSS, IHE)** | China (CDE) |
| --- | --- | --- | --- | --- | --- |
| Columbia (IETS) | Commonwealth of Australia  (ASERNIP-S, AHTA) | Denmark (DEFACTUM) | Egypt (CA-HTA) | Finland (FinCCHTA) | France (HAS, AP-HP) |
| **Germany (IQWiG)** | Ireland (HIQA) | Islamic Republic of Iran | Italy (UVT, RER, Agenas) | Japan (C2H) | Kazakhstan (SK-NRCHD) |
| Kingdom of Saudi Arabia | **Kingdom of Sweden (SBU)** | Malaysia (MaHTAS) | Netherlands (ZIN, ZonMw) | **Norway (NIPH)** | Peru (IETSI, DIGEMID ) |
| Poland (AOTMiT) | Portugal  (NAMHP) | Republic of Korea (NECA) | Slovak Republic (NIHO) | Singapore (ACE) | Spain (AETS, OSTEBA, IACS, AVALIA-T, AQuAS, AETSA, AETS) |
| **Switzerland (SFOPH)** | Tunisia (INESAS) | Ukraine (SEC) | United Arab Emirates | United Kingdom (NIHR, NICE, HIQA, HIS, HTW) | United States of America (AHRQ) |
| Uruguay (HAD) |  |  |  |  |  |

**Appendix 2: Overview of the 37 countries analyzed** (six were included, green)

Appendix 3

| Name of HTA report | Name of the agency | Publication date | Link (“main page” or “main report”) |
| --- | --- | --- | --- |
| Community Water Fluoridation Programs: A Health technology Assessment - Review of dental Caries and Other health Outcomes | Canadian Agency for Drugs and Technologies in Health (CADTH) | 01.02.2019 | [Community Water Fluoridation Programs: A Health Technology Assessment — Review of Dental Caries and Other Health Outcomes (fluoridealert.org)](https://fluoridealert.org/wp-content/uploads/cadth.dental-caries-other-health.2019.pdf) |
| Dental Amalgams Compared With Composite Resin | CADTH | 04.08.2017 | [Composite Resin Versus Amalgam for Dental Restorations: A Health Technology Assessment (cda-amc.ca)](https://www.cda-amc.ca/sites/default/files/pdf/ht0021_dental_amalgam_report_final.pdf) |
| e-Consult for Primary Care Clinician Access to Specialists | CADTH | 01.06.2022 | [HC0021-HT0030-e-consult-technology-review.pdf (cda-amc.ca)](https://www.cda-amc.ca/sites/default/files/hta-he/HC0021-HT0030-e-consult-technology-review.pdf) |
| Proton Beam Therapy for the Treatment of Cancer in Children and Adults | CADTH | 15.08.2017 | [Proton Beam Therapy for the Treatment of Cancer in Children and Adults: A Health Technology Assessment (nih.gov)](https://www.ncbi.nlm.nih.gov/books/NBK531691/pdf/Bookshelf_NBK531691.pdf) |
| Andwendungsvergleich bei Medikamenten gegen Knochenmetastasen | Federal Office of Public Health (FOPH) | 06.05.2022 | <https://www.bag.admin.ch/bag/de/home/versicherungen/krankenversicherung/krankenversicherung-leistungen-tarife/hta/hta-projekte/bonetargetingagents.html> |
| Behandlung einer nicht-erosiven gastroösophagealen Refluxerkrankung mit Protonenpumpeninhibitoren | FOPH | 17.06.2023 | <https://www.bag.admin.ch/bag/de/home/versicherungen/krankenversicherung/krankenversicherung-leistungen-tarife/hta/hta-projekte/protonenpumpeninhibitoren.html> |
| Beruhigende und schlaffördernde Medikamente zur Behandlung chronischer Schlafstörungen | FOPH | 19.01.2022 | <https://www.bag.admin.ch/bag/de/home/versicherungen/krankenversicherung/krankenversicherung-leistungen-tarife/hta/hta-projekte/sedativa.html> |
| CDK4/6 Inhibitoren bei fortgeschrittenem Brustkrebs | FOPH | 01.09.21 | <https://www.bag.admin.ch/bag/de/home/versicherungen/krankenversicherung/krankenversicherung-leistungen-tarife/hta/hta-projekte/cdk46inhibitoren.html> |
| Denosumab (Prolia) zur behandlung von Osteoporose | FOPH | 06.05.2022 | <https://www.bag.admin.ch/bag/de/home/versicherungen/krankenversicherung/krankenversicherung-leistungen-tarife/hta/hta-projekte/denosumab.html> |
| Hormontests bei einer vermuteten Schilddrüsenfunktionsstörung | FOPH | 11.11.2022 | <https://www.bag.admin.ch/bag/de/home/versicherungen/krankenversicherung/krankenversicherung-leistungen-tarife/hta/hta-projekte/schilddruesenhormontests.html> |
| Medikamente gegen Demenz bei Alzheimer und Parkinson | FOPH | 30.09.2022 | <https://www.bag.admin.ch/bag/de/home/versicherungen/krankenversicherung/krankenversicherung-leistungen-tarife/hta/hta-projekte/medikamentebeialzheimerdemenz.html> |
| Medizinischer Cannabis zur Behandlung verschiedener Beschwerden | FOPH | 18.04.2021 | <https://www.bag.admin.ch/bag/de/home/versicherungen/krankenversicherung/krankenversicherung-leistungen-tarife/hta/hta-projekte/medizinalcannabis.html> |
| Therapien mit Olmesartan bei Menschen mit dauerhaft hohem Blutdruck | FOPH | 08.06.2023 | <https://www.bag.admin.ch/bag/de/home/versicherungen/krankenversicherung/krankenversicherung-leistungen-tarife/hta/hta-projekte/olmesartan.html> |
| Vitamin-B12-Tests | FOPH | 11.06.2021 | <https://www.bag.admin.ch/bag/de/home/versicherungen/krankenversicherung/krankenversicherung-leistungen-tarife/hta/hta-projekte/vitaminb12tests.html> |
| Non-invasive prenatal testing (NIPT) for foetal sex determination | Norwegian Institute of Public Health (NIPH) | 13.12.2016 | <https://www.fhi.no/en/publ/2016/ikke-invasiv-prenatal-testing-nipt-for-kjonnsbestemmelse-av-foster.-metodev/> |
| Organ donation with the use of normothermic regional perfusion in patients who die after cardiac and respiratory arrest after withdrawal of life-sustaining treatment | NIPH | 17.12.2019 | <https://www.fhi.no/en/publ/2019/Organ-donation-with-the-use-of-normothermic-regional-perfusion/> |
| Continuity of Care | Swedish Agency for Health Technology Assessment and assessment of Social Service (SBU) | 19.08.2021 | <https://www.sbu.se/en/publications/sbu-assesses/continuity-of-care--a-systematic-review-and-assessment-of-medical-health-economic-and-social-aspects/> |
| Dementia - Caring, Ethics, Ethnical and Economical Aspects | SBU | 18.06.2008 | <https://www.sbu.se/en/publications/sbu-assesses/dementia/dementia--caring-ethics-ethnical-and-economical-aspects/> |
| Instruments for Suicide Risk Assessment | SBU | 23.09.2015 | <https://www.sbu.se/242e> |
| Medical and Psychological Methods for Preventing Sexual offences Against Children | SBU | 01.12.2011 | <https://www.sbu.se/en/publications/sbu-assesses/treatment-methods-for-child-molesters-or-those-at-risk-of-committing-sexual-offences-againt-children-/> |
| Organisational models for securing access to health and dental care services for children in out-of-home care | SBU | 13.06.2018 | <https://www.sbu.se/en/publications/sbu-assesses/organisational-models-for-securing-access-to-health-and-dental-care-services-for-children-and-young-adults-in-out-of-home-care/> |
| Risk and needs assessment regarding reoffending in adolescents | SBU | 20.11.2019 | <https://www.sbu.se/303e> |
| Traumatic shaking – The role of the triad in medical investigations of suspected traumatic shaking | SBU | 26.10.2016 | <https://www.sbu.se/255e> |
| Treatment of Women with diastasis recti | SBU | 15.03.2022 | <https://www.sbu.se/en/publications/sbu-assesses/treatment-of-women-with-diastasis-recti--a-hta-report/> |
| Angststörungen: Führt der ergänzende Einsatz der Eye Movement Desensitization and Reprocessing Therapie bei psychotherapeutischen Behandlungs- und Anwendungsformen zu besseren Ergebnissen? | Institute for Quality and Efficiency in Health Care (IQWiG) | 21.02.2020 | <https://www.iqwig.de/sich-einbringen/themencheck-medizin/berichte/ht17-05.html> |
| Empfängnisverhütung: Vergleich von Hormonspirale und Kupferspirale | IQWiG | 19.04.2023 | <https://www.iqwig.de/sich-einbringen/themencheck-medizin/berichte/ht21-05.html> |
| Fortgeschrittenes Lymphödem: Lassen sich durch nicht medikamentöse Verfahren die Symptome lindern? | IQWiG | 10.05.2022 | <https://www.iqwig.de/sich-einbringen/themencheck-medizin/berichte/ht19-01.html> |
| Halswirbelsäulensyndrom: Einfluss von Behandlungsdauer und - häufigkeit einer Physiotherapie auf den Behandlungserfolg | IQWiG | 03.07.2020 | <https://www.iqwig.de/sich-einbringen/themencheck-medizin/berichte/ht18-02.html> |
| Herzerkrankungen bei Kindern: Können Kinder durch eine psychologische Begleitung bei der Bewältigung ihrer Krankheit unterstütz werden? | IQWiG | 09.09.2022 | <https://www.iqwig.de/sich-einbringen/themencheck-medizin/berichte/ht20-02.html> |
| Hodenkrebs: Führt eine regelmäßige Früherkennungsuntersuchung für Männer ab 16 Jahren zu besseren Behandlungsergebnissen? | IQWiG | 03.07.2020 | <https://www.iqwig.de/sich-einbringen/themencheck-medizin/berichte/ht18-01.html> |
| Prostatakrebs: Führt die Anwendung der Fusionsbiopsie im Vergleich zur Anwendung üblicher diagnostischer Verfahren zu besseren Behandlungsergebnissen? | IQWiG | 12.03.2021 | <https://www.iqwig.de/sich-einbringen/themencheck-medizin/berichte/ht18-03.html> |
| Schmerzen bei Endometriose: helfen anstelle von Schmerzmedikamenten auch andere Verfahren? | IQWiG | 30.12.2021 | <https://www.iqwig.de/sich-einbringen/themencheck-medizin/berichte/ht19-02.html> |
| Soziale Isolation und Einsamkeit im Alter: Welche maßnahmen können einer sozialen Isolation vorbeugen oder entgegenwirken? | IQWiG | 30.11.2022 | <https://www.iqwig.de/sich-einbringen/themencheck-medizin/berichte/ht20-03.html> |
| Suizidale Krisen bei unipolarer depression: Welchen Einfluss haben nicht medikamentöse Maßnahmen auf deren Bewältigung | IQWiG | 30.10.2019 | <https://www.iqwig.de/sich-einbringen/themencheck-medizin/berichte/ht17-03.html> |
| Restless-Legs-Syndrom (unruhige Beine): Lassen sich durch nicht medikamentöse Verfahren die Symptome lindern? | IQWiG | 08.08.2023 | <https://www.iqwig.de/sich-einbringen/themencheck-medizin/berichte/ht21-04.html> |
| Demenz-Qualitätsregister: Kartierung von Registern zur Verbesserung der Qualität und Leistungserbringung | Austrian Institute for Health Technology Assessment (AIHTA) | 30.11.2020 | [HTA-Projektbericht_Nr.150.pdf (aihta.at)](https://eprints.aihta.at/1419/1/HTA-Projektbericht_Nr.150.pdf) |
| Molekulargenetische Diagnostik der Familiären Hypercholesterinämie | AIHTA | 30.11.2020 | [HTA-Projektbericht_Nr.130.pdf (aihta.at)](https://eprints.aihta.at/1281/1/HTA-Projektbericht_Nr.130.pdf) |
| Orale und parenterale Präexpositionsprophylaxe zur HIV-Prävention bei Risikopersonen | AIHTA | 17.04.2023 | [HTA-Projektbericht_Nr.152.pdf (aihta.at)](https://eprints.aihta.at/1436/1/HTA-Projektbericht_Nr.152.pdf) |
| Entscheidungsfindung an der Grenze der Lebensfähigkeit und Berufsethik bei neonatalogische Intensivstationen | Ludwig Boltzmann Institute for Health Technology Assessment (LBI-HTA) | 11.01.2018 | [HTA-Projektbericht_Nr.97b.pdf (aihta.at)](https://eprints.aihta.at/1148/1/HTA-Projektbericht_Nr.97b.pdf) |
| Screening mit nicht-invasiven pränatalen Tests auf fetale Trisomien | LBI-HTA | 09.02.2018 | [HTA-Projektbericht_Nr.103.pdf (aihta.at)](https://eprints.aihta.at/1153/1/HTA-Projektbericht_Nr.103.pdf) |

**Appendix 3: List of all 39 analyzed “full” HTA**

Appendix 4

|  | Clinical effectiveness & safety | Costs and economic evaluation | Ethical  aspects | Legal  aspects | Social  aspects | Organizational  aspects |
| --- | --- | --- | --- | --- | --- | --- |
| **Canada** (n = 4) | **100%** | **100%** | **100%** | 0% | 50% | **100%** |
| **Switzerland** (n = 10) | **100%** | **100%** | **100%** | **100%** | 90% | 90% |
| **Germany** (n = 10) | **100%** | **100%** | **100%** | **100%** | **100%** | **100%** |
| **Austria** (n = 5) | 80% | 60% | **100%** | 40% | 40% | 40% |
| **Sweden** (n = 8) | 75% | 88% | **100%** | 13% | 38% | 0% |
| **Norway** (n = 2) | **100%** | 50% | **100%** | 50% | 0% | 0% |

**Appendix 4: Domains included in the 39 HTA reports included for analysis**

Appendix 5

| **Approach** | | **n=** | **Explanation** |
| --- | --- | --- | --- |
| **Socratic approach**  (unspecified: n = 1; specified n = 13, see below) | | **14** | Approach that follows the original Socratic method (maieutic) and only asks questions to stimulate one's own thinking about ethical aspects in a technology. |
| - (Hofmann et al. 2014) | | 11/14 | Specific Socratic-oriented approach that provides 33 questions. |
| - (Stich 2018) | | 1/14 | Specific list of 14 questions for social and cultural aspects in health technology assessment. |
| - (Helsedirektoratet 2012) | | 1/14 | The method is based on the Hofmann catalogue (see above) and consists of six procedural steps (e.g. identification of stakeholders, conduction of a literature review). Additionally, the guideline should specify which value base has been applied and which adaptations may be relevant for patients with different values and preferences. |
| **Principlism approach**  (unspecified: n = 1; specified n = 8, see below) | | **9** | Approach which starts from ethical principles of “middle range” that are *prima facie* valid; only in the case-related balancing and weighting does it become clear which principle is to be followed. The principles do not claim to cover morality as a whole but only the area of action for which the approach is intended (e.g. medicine). One report, for example, used the following principles: beneficence, respect of autonomy, fairness/equity and stewardship (Beauchamp and Childress 2019; Kim et al. 2017). |
| - (Marckmann 2018) | | 4/9 | Specific principlism approach that starts from five principles intended for public health interventions: beneficence, non-maleficence, respect of autonomy, justice and maximising benefits. |
| - (Beauchamp and Childress 2019) | | 3/9 | Classical approach to principlism (particularly for clinical ethics), based on four principles: beneficence, non-maleficence, respect of autonomy and justice. |
| - (Childress and Bernheim 2013) | | 1/9 | Specific principlism approach for public heath ethics, consisting of nine principles: beneficence, non-maleficence, utility, procedural justice, respect of autonomy, privacy and confidentiality, keeping promises and commitments, transparency and trust. |
| **EUnetHTA core model** (EUnetHTA 2016) | | **4** | The ethics domain includes six different topics (benefit-harm balance, autonomy, respect for persons, justice and equity, legislation and ethical consequences of the HTA). Further ethical considerations should be taken into account when choosing what technologies to assess and when planning to conduct the assessment. |
| **SBU Guidelines** (Heintz et al. 2015) | | **2** | The framework for systematic identification of ethical aspects of health technologies consists of 12 items with sub-questions, short explanations and a concluding overall summary. The items are organized into four different themes: the effects of the intervention on health, its compatibility with ethical norms, structural factors with ethical implications and long-term ethical consequences of using the intervention. |
| **Deontological approach** | | **1** | Approach that assumes that actions can be intrinsically morally right or wrong; in the HTA context, this usually refers to approaches that assume duties or specific norms. |
| **Integrate HTA Framework** (Lysdahl et al. 2016) | | **1** | The ethics guidance provides a stepwise procedure for addressing ethical aspects in the assessment of HTA (e.g. assessing the complexity of the technology, selecting the best type of ethical approach for this particular technology) |
| References | **Hofmann**, B., et al., *Harmoniziation of Ethics in Health Technology Assessment: a revision of the Socratic approach.* Intern. Journal of Technology Assessment in Health Care, 2014. **30**(1): p. 3-9.  **Stich**, A.K., *Soziale und kulturelle Aspekte im Health Technology Assessment - Eine Methodenübersicht und Methodenanwendung am Beispiel der medikamentösen Behandlung mit Methylphenidat von Kindern und Jugendlichen mit Aufmerksamkeitsdefizit-/Hyperaktivitätsstörung*. 2018, Bielefeld: Universität Bielefeld.  **Helsedirektoratet**, *Veilder for utvukling av kunnskapsbaserte retningslinjer*, C. Hodt-Billington, Editor. 2012: Oslo.  **Marckmann**, G., Ethische Bewertung von Public Health Maßnahmen. Methodische Grundlagen und praktische Anwendung, in Medizin und öffentliche Gesundheit. Konzepte, Akteure, Perspektiven, H.-P. Schmiedebach, Edit. 2018, De Gruyter: Oldenburg.  **Beauchamp**, T.L. and J. Childress, *Principles of Biomedical Ethics*. 8th ed. 2019, New York: Oxford University Press.  **Childress**, J.F. and R.G. Bernheim, *Introduction: A framework for public health ethics*, in *Essentials of public health ethics*, R.G. Bernheim, et al., Edit. 2013, Jones and Bartlett: Burlington, MA.  **EUnetHTA**, *Work Package 8. HTA Model version 3.0*. 2016. Available from: <https://www.eunethta.eu/hta-core-model/>.  **Heintz**, E., et al., *Framework for systematic identification of ethical aspects of healthcare technologies: the SBU approach.* International Journal of Technology Assessment in Health Care, 2015. **31**(3): p. 124-130.  **Lysdahl**, K., et al., *Ethical analysis in HTA of complex health interventions.* BMC Med Ethics, 2016. **17**(6). | | |

**Appendix 5: Theoretical and procedural approaches for processing the ethics domain in an HTA**
